# Autopsy analyses in acute exacerbation of idiopathic pulmonary fibrosis

**DOI:** 10.1186/s12931-014-0109-y

**Published:** 2014-09-01

**Authors:** Keishi Oda, Hiroshi Ishimoto, Sohsuke Yamada, Hisako Kushima, Hiroshi Ishii, Tomotoshi Imanaga, Tatsuhiko Harada, Yuji Ishimatsu, Nobuhiro Matsumoto, Keisuke Naito, Kazuhiro Yatera, Masamitsu Nakazato, Jun-ichi Kadota, Kentaro Watanabe, Shigeru Kohno, Hiroshi Mukae

**Affiliations:** Department of Respiratory Medicine, University of Occupational and Environmental Health, 1-1, Iseigaoka, Yahatanishiku, Kitakyushu City, Fukuoka 807-8555 Japan; Department of Pathology and Cell Biology, University of Occupational and Environmental Health, 1-1, Iseigaoka, Yahatanishiku, Kitakyushu City, Fukuoka 807-8555 Japan; Department of Respiratory Medicine and Infectious Diseases, Oita University Faculty of Medicine, 1-1 Idaigaoka, Hasama-machi, Oita, 879-5593 Japan; Department of Respiratory Medicine, Fukuoka University School of Medicine, 7-45-1, Nakakuma, Jonanku, Fukuoka 814-0180 Japan; Department of Respiratory Medicine, Steel Memorial Yawata Hospital, 1-1-1, Harunomachi, Yahatahigashiku, Kitakyushu City, Fukuoka 805-8508 Japan; Second Department of Internal Medicine, Nagasaki University School of Medicine, 1-7-1 Sakamoto, Nagasaki, 852-8501 Japan; Neurology, Respirology, Endocrinology and Metabolism, Internal Medicine, Faculty of Medicine, University of Miyazaki, 889-1692 Miyazaki, Japan

**Keywords:** Acute exacerbation, Idiopathic pulmonary fibrosis, Autopsy, Diffuse alveolar damage

## Abstract

**Background:**

Acute exacerbation of idiopathic pulmonary fibrosis (AE-IPF) is associated with high mortality. However, few studies have so far reviewed analyses of autopsy findings in patients with AE-IPF.

**Methods:**

We retrospectively reviewed 52 consecutive patients with AE-IPF who underwent autopsies at five university hospitals and one municipal hospital between 1999 and 2013. The following variables were abstracted from the medical records: demographic and clinical data, autopsy findings and complications during the clinical course until death.

**Results:**

The median age at autopsy was 71 years (range 47–86 years), and the subjects included 38 (73.1%) males. High-dose corticosteroid therapy was initiated in 45 (86.5%) patients after AE-IPF. The underling fibrotic lesion was classified as having the usual interstitial pneumonia (UIP) pattern in all cases. Furthermore, 41 (78.8%) patients had diffuse alveolar damage (DAD), 15 (28.8%) exhibited pulmonary hemorrhage, nine (17.3%) developed pulmonary thromboembolism and six (11.5%) were diagnosed with lung carcinoma. In addition, six (11.5%) patients developed pneumothorax prior to death and 26 (53.1%) developed diabetes that required insulin treatment after the administration of high-dose corticosteroid therapy. In addition, 15 (28.8%) patients presented with bronchopneumonia during their clinical course and/or until death, including fungal (seven, 13.5%), cytomegalovirus (six, 11.5%) and bacterial (five, 9.6%) infections.

**Conclusions:**

The pathological findings in patients with AE-IPF represent not only DAD, but also a variety of pathological conditions. Therefore, making a diagnosis of AE-IPF is often difficult, and the use of cautious diagnostic approaches is required for appropriate treatment.

## Introduction

Idiopathic pulmonary fibrosis (IPF) is a chronic, progressive, fibrosing form of interstitial pneumonia with a median survival after diagnosis of three to five years [1-4]. Acute exacerbation of IPF (AE-IPF) has an extremely poor prognosis and is believed to occur in 5-10% of patients with IPF annually [5,6]. In 1993, AE-IPF was first described in a case report by Kondoh et al. as acute clinical deterioration in three IPF patients in the absence of an identified infection [7]. The American Thoracic Society and European Respiratory Society subsequently introduced the notion of AE-IPF in 2002 [8], and Collard and colleagues focused on establishing a worldwide consensus for AE-IPF in 2007 [9]. This consensus statement is currently the most widely used definition of AE-IPF, having been used in several clinical studies and being referred to in the 2011 IPF guidelines [10].

Lung tissue derived from patients with IPF shows a characteristic histopathological pattern known as usual interstitial pneumonia (UIP), which includes the presence of fibroblastic foci. The pathological findings of AE-IPF comprise diffuse alveolar damage (DAD) superimposed on underlying UIP; this is the most commonly described finding on surgical lung biopsies [9,11,12]. On the other hand, several studies have reported the detection of organizing pneumonia (OP) without evidence of organizing DAD or extensive fibroblastic foci [13,14]. In addition, although previous reports have focused on the postmortem pathological findings of patients with IPF [15,16], no studies have focused on the characteristics of “acute exacerbation”, and the pathological findings and clinical manifestations of AE-IPF remain only partially understood.

The current study is a retrospective review of an autopsy series designed to describe and evaluate the pathological findings, including concomitant and infectious diseases, observed during the clinical course until death in patients with AE-IPF.

## Methods

### Patients

A total of 52 patients with AE-IPF who underwent autopsies at five university hospitals and one municipal hospital between January 1, 1999 and December 31, 2013 were identified based on medical records. The Ethics Committee of University of Occupational and Environmental Health in Kitakyushu, Japan approved this study (approval number H26-12, April 3, 2014), with a waiver for informed consent due to the retrospective study design. AE-IPF was defined according to a previous report [9] and included the following items: [1] a previous or concurrent diagnosis of idiopathic pulmonary fibrosis; [2] unexplained worsening or new development of dyspnea within 30 days; [3] high-resolution computed tomography (HRCT) findings showing new sites of bilateral ground-glass opacity; [4] abnormalities and/or consolidation superimposed on a background of a reticular or honeycomb pattern consistent with the UIP pattern; [5] no evidence of pulmonary infection on an endotracheal aspirate or bronchoalveolar lavage (BAL) sample and [6] the exclusion of left heart failure, pulmonary embolism and other identifiable causes of acute lung injury. Regarding infectious causes in the current study, we carefully excluded possible infections based on the findings of a sputum examination, laboratory tests and physical examinations, although we did not perform endotracheal aspiration or BAL in all cases due to the presence of severe hypoxemia. In addition, we attempted to rule out pulmonary embolism as far as possible using enhanced computed tomography. The following variables were abstracted from the medical records: demographic data (age, sex, smoking history, symptoms, comorbidities and prior treatment for IPF), clinical data (hospitalization before death, laboratory results and HRCT findings) and the autopsy findings, including infectious causes.

### Autopsy findings in the patients with AE-IPF

All autopsied lung materials were fixed in 10% formalin for more than seven days. At least one tissue block was prepared from the gross AE-IPF lesions in each lobe, and the tissue blocks were embedded in paraffin. Sections (4 μm thick) were cut and stained with hematoxylin and eosin (H&E) using standard procedures. The pathological diagnosis of UIP was made according to the current guidelines [10]. The criteria for DAD were the presence of hyaline membranes in addition to at least one of the following findings: alveolar type I cell or endothelial cell necrosis, edema, organizing interstitial fibrosis or prominent alveolar type II cell proliferation [15]. We also performed phosphotungstic acid hematoxylin staining for fibrin in patients without a hyaline membrane on H&E staining. OP was pathologically defined as the presence of buds of granulation tissue in the distal air spaces progressing from fibrin exudates to loose collagen-containing fibroblasts [16]. Alveolar hemorrhage was diagnosed in cases involving acute hemorrhage in the alveoli and airways as well as the presence of macrophages (i.e., siderophages) that stained positively for hemosiderin with Berlin-blue stain. Right ventricular hypertrophy was defined as a right ventricular free wall thickness of ≥ 5 mm [17].

### Screening for infectious diseases in the autopsied lungs

Four-micrometer-thick sections were cut from the lung tissue blocks and stained with H&E for the histopathological examinations in addition to Periodic acid-Schiff (PAS) stain, Grocott's methenamine silver stain and Gram stain to identify any infectious pathogens in the presence of bronchopneumonia. Bronchopneumonia (i.e., concomitant infection) was conclusively diagnosed based on histopathological evidence, such as neutrophil infiltration, fibrinopurulent exudate accumulation or abscess formation in the broncho-bronchioloalveolar space. For immunohistochemical detection of cytomegalovirus (CMV) antigens, the sections were incubated with a mouse monoclonal anti-CMV antibody (DAKO, Glostrup, Denmark; diluted 1:20) for 30 minutes. Secondary antibody peroxidase-linked polymers were then applied, and the sections were incubated with a solution consisting of 20 mg of 3.3’-diaminobenzidine tetrahydrochloride, 65 mg of sodium azide and 20 ml of 30% H_2_O_2_ in 100 ml of Tris–HCl (50 mM, pH 7.6). After counterstaining with Meyer’s hematoxylin, the sections were observed under a light microscope.

## Results

### Patient characteristics

A total of 52 patients with AE-IPF underwent autopsies during the study period. The demographics of the patients are reported in Table 1. All patients had a known diagnosis of IPF in addition to either unexplained worsening or the development of dyspnea within 30 days and were hospitalized at the time of death. A large majority of patients experienced acute episodes of AE-IPF, including 41 (78.8%) patients with coughing and 22 (42.3%) patients with a fever. Only one patient was diagnosed with pulmonary hypertension prior to AE-IPF, whereas 12 patients were additionally diagnosed with pulmonary hypertension at the onset of AE-IPF based on the findings of right heart catheterization and/or transthoracic echocardiography [18]. The median survival period for the patients with AE-IPF was approximately 29 days (range: 1 to 134 days) from admission.

**Table 1:** **The clinical findings of the 52 patients with acute exacerbation of idiopathic pulmonary fibrosis on admission**

**Characteristic**	**Data**
Patient, No. (male/female)	52 (38/14)
Age, years old, mean (range)	71.1 (47–86)
Smoking (current/former/never)	9/22/21
Symptoms	
Dyspnea	52 (100%)
Cough	41 (78.8%)
Fever	22 (42.3%)
Hemoptysis	2 (3.8%)
Comorbidities	
Diabetes	18 (34.6%)
Chronic heart failure	12 (23.1%)
Cancer	7 (13.5%)
Pulmonary hypertension	1 (1.9%)
Prior treatment(s)	
Corticosteroid monotherapy	13 (25.0%)
Corticosteroid plus immunosuppressive agent	12 (23.1%)
Pirfenidone	5 (9.6%)
Home oxygen therapy	15 (28.8%)
Blood sample findings	
WBC, /μl, mean (range)	11434 (4000–21300)
CRP, mg/dl, mean (range)	9.36 (0.1-25.9)
LDH, IU/L, mean (range)	479.2 (138–4135)
KL-6, U/ml, mean (range)	1855 (507–7280)
SP-D, ng/ml, mean (range)	545 (144–2500)

Data are presented as the n and means.

Nearly half of the patients had received corticosteroid therapy before AE-IPF. The therapeutic regimens for AE-IPF are shown in Table 2. Forty-five (86.5%) patients had received high-dose corticosteroid therapy, 43 (82.7%) had received antibacterial drugs and 27 (51.9%) were on mechanical ventilation due to hypoxemia. Sulfamethoxazole/trimethoprim was used to prevent pneumocystis pneumonia in 21 (40.4%) patients. Six (11.5%) patients developed pneumothorax prior to death and 26 (57.8%) developed insulin-dependent diabetes after receiving high-dose corticosteroid therapy.

**Table 2:** **The therapeutic regimen use for the AE-IPF**

**Treatment**	**No. (%)**
High-dose corticosteroids	45 (86.5)
Immunosuppressive agents	15 (28.8)
Sivelestat sodium	17 (32.7)
PMX-DHP	7 (13.5)
Anticoagulant therapy	12 (23.1)
Mechanical ventilation	27 (51.9)
NPPV	4 (7.7)
Antibacterial drug	43 (82.7)
Carbapenems	15 (28.8)
Quinolones	12 (23.1)
Penicillin-based drugs	4 (7.7)
Cephems	5 (9.6)
Others	7 (13.5)

*Abbreviations:* *AE-IPF* Acute exacerbation of idiopathic pulmonary fibrosis, *PMX-DHP* Polymyxin-B direct hemoperfusion, *NPPV* Noninvasive positive pressure ventilation.

### Pathological findings

**Figure 1:**
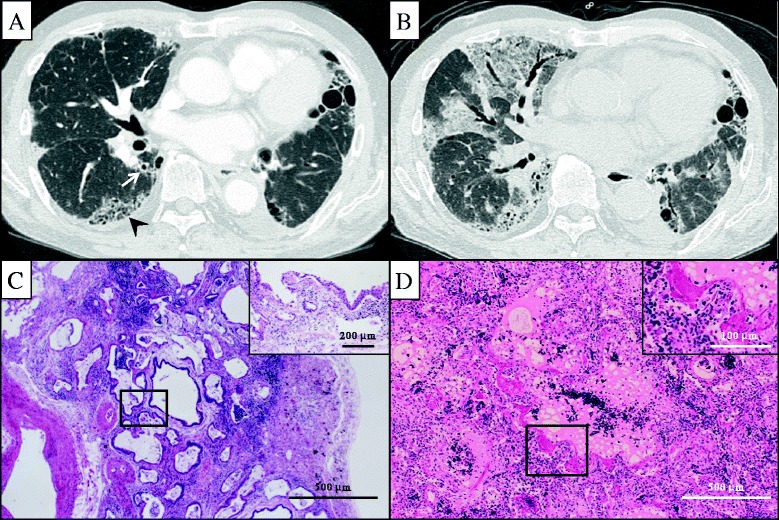
**A 78-year-old male with acute exacerbation of idiopathic pulmonary fibrosis (AE-IPF).** High resolution computed tomography (HRCT) images at the initial diagnosis of IPF showed subpleural-predominant interstitial fibrosis, traction bronchiectasis (arrow) and honeycombing (arrowhead) **(A)**. HRCT images at the onset of acute exacerbation (12 months after the initial diagnosis) showed diffuse areas of ground glass attenuations superimposed on underlying fibrotic opacities **(B)**. The underling fibrotic lesions were classified as the UIP pattern, including dense interstitial fibrosis with focal squamous or bronchial metaplasia (inset), alternating with only bland-looking alveolar walls (H&E, ×100) **(C)**. DAD with hyaline membranes (inset) superimposed on a background fibrotic and edematous lung with several fibroblastic foci (H&E, ×100) **(D)**.

**Figure 2:**
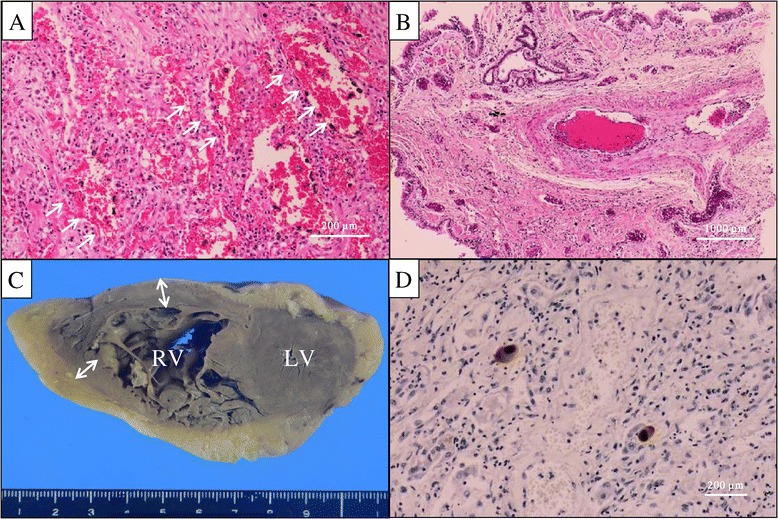
**The histopathological findings of the lung specimens obtained at autopsy.** The microscopic findings of the lung showed diffuse alveolar hemorrhage (arrows) without any specific evidence of vasculitis **(A)** or pulmonary thromboembolism **(B)** (H&E, ×10). The macroscopic findings of the heart showed overt right ventricular hypertrophy, confirmed by an increased (≥5 mm) right ventricular wall thickness (double-headed arrows) **(C)**. Immunohistochemical staining of an inflamed lung tissue specimen revealed a number of specific cytomegalovirus-positive cells (proliferating type II pneumocytes) **(D)**.

The mean duration between death and autopsy was 286 minutes (range: 60 to 990 minutes). Overall, the median weights of the right and left lungs were 658 g (range: 320 to 1,330 g) and 552 g (range: 260 to 1,000 g), respectively. These median weights were remarkably heavier than the standard lung weights reported for Japanese males (right and left lung weights: 474 and 404 g, respectively) [19]. The underlying pulmonary fibrotic lesion was classified as exhibiting the UIP pattern in all cases. Forty-one (78.8%) patients had DAD (Figure 1A-D), 15 (28.8%) patients had pulmonary hemorrhage (Figure 2A), nine (17.3%) patients had pulmonary thromboembolism (Figure 2B) and six (11.5%) patients had lung carcinoma (Table 3). Only two of the 15 AE-IPF patients with pulmonary hemorrhage received anticoagulant therapy.

**Table 3:** **The autopsy findings of patients with AE-IPF**

**Pathological findings**	**No. (%)**
UIP pattern	52 (100)
Diffuse alveolar damage	41 (78.8)
Alveolar hemorrhage	15 (28.8)
Organizing pneumonia	1 (1.9)
Pulmonary thromboembolism	9 (17.3)
Lung cancer	6 (11.5)
Bronchopneumonia	15 (28.8)
Bacterial infection	6 (11.5)
Fungal infection	7 (13.5)
Cytomegalovirus infection	6 (11.5)
Extrapulmonary findings	
Gastrointestinal hemorrhage	13 (25.0)
Right ventricular hypertrophy	18 (34.6)

*Abbreviations:* *AE-IPF* Acute exacerbation of idiopathic pulmonary fibrosis, *UIP* Usual interstitial pneumonia.

**Figure 3:**
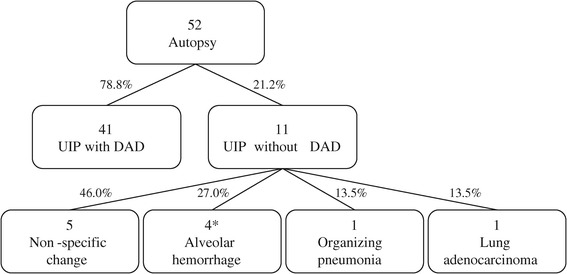
**A flow diagram showing the pathological findings of the patients with AE-IPF.** *One patient showed pulmonary thromboembolism with alveolar hemorrhage.

The pathological findings in the 11 patients with AE-IPF who met the 2007 criteria but did not have DAD were indicative of UIP alone (n = 5), alveolar hemorrhage (n = 3), pulmonary thromboembolism (n = 1), OP (n = 1) and lung adenocarcinoma (n = 1) (Figure 3). There were no significant differences in any of the patient characteristics between the UIP with DAD and UIP without DAD groups on admission. In addition, there were no correlations or significant differences between the degree of pulmonary hypertension and histological variation. Among the 52 patients with AE-IPF, 13 (25.0%) exhibited gastrointestinal hemorrhage and 18 (34.6%) displayed right ventricular hypertrophy (Figure 2C) as extrapulmonary findings.

### Infectious causes

Of the 52 autopsies performed, death was attributed to bronchopneumonia in 15 (28.8%) patients (Table 4). In these cases, the pulmonary infectious lesions were not diagnosed until autopsy. The causes of infection included fungal infection in seven cases (13.5%), CMV infection (Figure 2D) in six cases (11.5%) and bacterial infection in five cases (9.6%). One patient with pulmonary aspergillosis died on the first day after AE-IPF; however, the postmortem pathological findings of the lungs in this case primarily showed a DAD pattern, with only slight bronchopneumonia induced by aspergillosis. Gram staining demonstrated the infectious bacteria to be Gram-negative rods and Gram-positive cocci. Pneumocystis pneumonia was not detected in any patient in the present study. All patients with AE-IPF, except one, had received high-dose corticosteroid therapy and/or immunosuppressive agents. There were also no significant differences in the time interval between the diagnosis of AE-IPF and death based on whether the patient was diagnosed with infectious disease (29.8 vs 31.1 days, p = 0.87).

**Table 4:** **The characteristics of the 15 patients with positive results for infectious causes**

**Patient, No.**	**Age**	**Sex**	**Time from AE-IPF to death (days)**	**Prior treatment for IPF**	**Treatment for AE-IPF**	**Mechanical ventilation**	**Bacteria**	**Virus**	**Fungus**
1	74	M	1	None	None	No	-	-	*Aspergillus* species
2	68	M	8	CS	High dose corticosteroids, CPA	Yes	-	CMV	-
3	66	M	10	None	High dose corticosteroids	Yes	-	-	*Aspergillus* species
4	67	M	12	None	High dose corticosteroids	Yes	GPC	-	-
5	76	F	13	CS	High dose corticosteroids, CPA	Yes	-	-	*Candia albicans*
6	78	M	15	None	High dose corticosteroids	No	GNR	CMV	-
7	68	M	19	None	High dose corticosteroids, CPA	Yes	GPC	-	-
8	83	M	19	CS, CsA	High dose corticosteroids, CsA	No	-	-	*Aspergillus* species
9	71	M	20	CS	High dose corticosteroids	Yes	-	-	*Aspergillus* species
10	68	M	22	None	High dose corticosteroids	Yes	-	CMV	-
11	81	M	35	CS	High dose corticosteroids, CPA	No	-	-	*Aspergillus* species
12	68	F	38	None	High dose corticosteroids, CPA	Yes	-	CMV	-
13	59	M	41	CS, CsA	High dose corticosteroids	No	GPC, GNR	CMV	-
14	76	M	58	None	High dose corticosteroids, CPA	Yes	-	CMV	-
15	80	M	122	CS	High dose corticosteroids	No	GNR	-	*Aspergillus* species

*Abbreviations:* *AE-IPF* Acute exacerbation of idiopathic pulmonary fibrosis, *CS* Corticosteroid, *CPA* Cyclophosphamide, *CsA* Cyclosporine, *GPC* Gram-positive cocci, *GNR* Gram-negative rods, *CMV* Cytomegalovirus.

## Discussion

In this study, we retrospectively analyzed the autopsy findings of AE-IPF and clarified that AE-IPF exhibits a variety of pathological findings in addition to DAD. Moreover, the AE-IPF patients were diagnosed with various infectious diseases and complications during their clinical course after AE-IPF.

DAD has been reported to be the focal pathological finding of AE-IPF [9]. DAD also accounted for many cases of AE-IPF in the present investigation, involving a mixture of pulmonary hemorrhage, pulmonary thromboembolism and OP. However, DAD was not observed in all cases. In particular, pulmonary hemorrhage was a representative pathological finding in the AE-IPF patients without DAD. Pulmonary hemorrhage is rarely encountered during the course of IPF [20-22], and it is difficult to make a premortem diagnosis in actual clinical practice without performing BAL. Nevertheless, careful attention is required in such cases, as the use of BAL in IPF patients may increase the risk of acute exacerbation [23]. Moreover, pulmonary thromboembolism frequently occurs as a complication during the course of IPF [24] and can be an important differential diagnosis of AE-IPF.

Regarding acute respiratory distress syndrome (ARDS), in which the diagnostic criteria are based on clinical findings, in the same manner as AE-IPF, Patel S.R. et al. reported that they investigated the pathological findings of 57 cases satisfying the diagnostic criteria for ARDS via open-lung biopsy, with DAD observed in 23 patients (40.3%) [25]. On the other hand, another study reported specific infections in eight patients (14.0%), diffuse alveolar hemorrhage in five patients (8.8%) and bronchiolitis obliterans organizing pneumonia in five patients (8.8%). Moreover, Esteban A. et al. found that, although DAD was observed in 112 of 127 autopsy cases satisfying the diagnostic criteria for ARDS, the following diseases were observed in patients without DAD, in order of descending prevalence: pneumonia, pulmonary hemorrhage, pulmonary edema and pulmonary embolism [26]. The findings of such diverse pathological features are very similar to the results of the present study. To date, very few investigations have been carried out regarding the pathological findings of AE-IPF, and the present study is the first report to summarize the features of autopsy cases involving the “acute exacerbation” of IPF. In this study, although the diagnosis was made while checking the findings of AE-IPF against the diagnostic criteria, in the same manner as diagnosing ARDS, not all of the patients with AE-IPF exhibited DAD, namely only 78.8% of the AE-IPF patients demonstrated DAD. Moreover, a significant number of patients who satisfied the diagnostic criteria for AE-IPF did not have remarkable DAD (11/52, 21.2%). In addition, we found no findings associated with acute fibrinous or organizing pneumonia in the AE-IPF patients without DAD [27]. In these cases, genuine respiratory failure accompanied the progression of pulmonary fibrosis, which may constitute the natural course of IPF. Notably, the results of the present study are supported by the findings of a previous report in which only extensive fibroblastic foci were observed as a pathological finding of an acute pattern [13]. The incidence of gastrointestinal hemorrhage and right ventricular hypertrophy, both of which are extrapulmonary findings, was 25.0% and 34.6%, respectively. Furthermore, the effects of hypoxic, physical and psychosomatic stress [28] and high-dose corticosteroid therapy [29] must be considered with respect to gastrointestinal hemorrhage. Right ventricular hypertrophy may be caused by pulmonary hypertension [30] at the end stage of AE-IPF. Moreover, the relationship between IPF and pulmonary hypertension is important, and Judge and colleagues recently reported that pulmonary hypertension is associated with acute disease exacerbation as well as poor survival [31]. Therefore, it is necessary to monitor the potential for pulmonary hypertension in patients with IPF.

Although there is no established standard therapy for AE-IPF [10], high-dose corticosteroid therapy [11,12,14,32-34] and immunosuppressive agents [35,36] are commonly used in clinical practice. However, a previous study [37] suggested that these drugs increase the rates of infectious complications. In addition to the immunodeficient state caused by corticosteroids and/or immunosuppressants, the application of intensive antimicrobial treatment following the acute exacerbation of pulmonary infection may have affected the results of the present study. Regardless, similar to recently published data [38] from a study of gene expression profiling of patients with AE-IPF, our data demonstrated that an infectious etiology may not be the main cause of AE-IPF. In fact, all patients underwent treatment with high-dose corticosteroids and/or immunosuppressive agents, excluding only one patient, with mechanical ventilation carried out in more than half of the AE-IPF patients. Moreover, the pulmonary infections detected in the present study may have affected, at least in part, the occurrence of respiratory failure leading to death, although there were no significant differences in the time interval between the diagnosis of AE-IPF and death based on the presence of infectious disease. Therefore, in addition to the detection of pathological findings based on DAD, AE-IPF eventually causes respiratory failure due to the effects of accompanying infectious diseases, with patients thus exhibiting a variety of histopathological features. The present investigation was carried out among autopsy cases only; therefore, the efficacy of high-dose corticosteroid therapy against AE-IPF cannot be debated based on our results. However, taking into consideration the fact that many patients with diabetes and pneumothorax following acute disease exacerbation underwent high-dose corticosteroid therapy, such therapy should be carefully administered based on the tolerability and efficacy of the treatment in conjunction with multidisciplinary therapies. Physicians should therefore be aware of the appropriate therapeutic strategies when treating patients with complications induced by treatment for AE-IPF. Furthermore, the results of the present study indicate that it is difficult to distinguish acute exacerbation from other disorders, even if the patient meets the diagnostic criteria for AE-IPF. Given the results of this study, it is very important for clinicians to be alert to the possibility of other treatable disorders, such as infectious diseases, as no effective treatment regimen for AE-IPF has been established to date.

There are several limitations associated with this study. First, only patients who died and underwent autopsy were included; therefore, the results may differ based on the status of onset of AE-IPF. In other words, no patients who survived after AE-IPF were included, and the present findings thus do not reflect all aspects of the entity of AE-IPF. Second, we did not perform endotracheal aspiration or bronchoalveolar lavage due to the presence of severe hypoxemia in all cases. However, we carefully excluded patients based on the findings of sputum, laboratory and physical examinations. In addition, a previous study reported that the features of clinically suspected acute exacerbation diagnosed based on the radiological and clinical course are similar to those of acute exacerbation diagnosed based on the results of several intensive examinations, including BAL [39]. Third, this study was carried out jointly across multiple facilities, primarily university hospitals, including only Japanese IPF patients. Hence, potential biases, including racial and institutional selection, must be considered when interpreting the results. Finally, 52% of the patients with AE-IPF in the present study required mechanical ventilation on admission, which suggests that these patients had a relatively more severe condition. Therefore, the results of this study may not be extrapolated to all cases of AE-IPF.

## Conclusions

The pathological findings of AE-IPF include DAD as well as a variety of pathological conditions, and making a definitive diagnosis of AE-IPF is difficult. Patients with AE-IPF therefore are at risk of death due to the occurrence of several complications during their clinical course, including the effects of treatment with high-dose corticosteroid therapy. In patients with AE-IPF, it is very important to treat the disease by monitoring the patient’s condition and timely intervening with appropriate treatment.

## Abbreviations

IPF: Idiopathic pulmonary fibrosis
AE-IPF: Acute exacerbation of idiopathic pulmonary fibrosis
UIP: Usual interstitial pneumonia
DAD: Diffuse alveolar damage
OP: Organizing pneumonia
HRCT: High-resolution computed tomography
BAL: Bronchoalveolar lavage
H&E: Hematoxylin and eosin
PAS: Periodic acid-Schiff
CMV: Cytomegalovirus
RV: Right ventricular
LV: Left ventricular
ARDS: Acute respiratory distress syndrome
